# Residual DCIS Versus No Residual Breast Tumor After Neoadjuvant Chemotherapy in ypN0 Breast Cancer: A Retrospective Clinical, MRI, and Survival Comparison

**DOI:** 10.3390/cancers18213491

**Published:** 2026-10-29

**Authors:** Özlem Mermut, Berrin Yalçın, Rıza Umar Gürsu, Esra Arslan, Semiha Battal Havare, Emine Sevcan Ata, Merve Büşra Cengiz Gökmen, Didem Can Trabulus

**Affiliations:** 1 Department of Radiation Oncology, University of Health Sciences Turkey, Istanbul Training and Research Hospital, 34098 Istanbul, Turkey; 2 Department of Medical Oncology, University of Health Sciences Turkey, Istanbul Training and Research Hospital, 34098 Istanbul, Turkey; 3 Department of Nuclear Medicine, University of Health Sciences Turkey, Istanbul Training and Research Hospital, 34098 Istanbul, Turkey; 4 Department of Pathology, University of Health Sciences Turkey, Istanbul Training and Research Hospital, 34098 Istanbul, Turkey; 5 Department of Radiology, University of Health Sciences Turkey, Istanbul Training and Research Hospital, 34098 Istanbul, Turkey; 6 Department of General Surgery, University of Health Sciences Turkey, Istanbul Training and Research Hos pital, 34098 Istanbul, Turkey; 7 Department of General Surgery, School of Medicine, Bahçeşehir University, 34734 Istanbul, Turkey

**Keywords:** breast cancer, neoadjuvant chemotherapy, residual ductal carcinoma in situ, ypTis, ypT0, MRI

## Abstract

**Background/Objectives:** The prognostic significance of residual ductal carcinoma in situ (ypTis) compared with no residual tumor in the breast (ypT0) after neoadjuvant chemotherapy (NAC) remains uncertain. We compared clinicopathological, MRI, treatment, and survival outcomes between these groups. **Methods:** This single-center retrospective study included consecutive patients with stage II–III breast cancer who achieved ypN0 and ypT0/is status after NAC and definitive surgery between 2019 and 2023. Univariable and multivariable logistic regression analyses were performed to identify factors associated with ypTis. DFS and OS were assessed using Kaplan–Meier analysis and log-rank tests. **Results:** Among 175 patients, 139 (79.4%) had ypT0 and 36 (20.6%) had ypTis. Median follow-up was 51 months. Clinical T-stage distribution differed significantly between groups (*p* = 0.010), with cT4 disease in 13.9% of ypTis versus 2.2% of ypT0 patients. HER2 positivity and MRI-detected satellite foci were more frequent in the ypTis group. In multivariable analysis, HER2 positivity (OR 2.50, 95% CI 1.05–5.93; *p* = 0.038), and MRI-detected satellite foci (OR 2.41, 95% CI 1.12–5.16; *p* = 0.024) were associated with ypTis. Local recurrence occurred in 3/36 (8.3%) ypTis and 1/139 (0.7%) ypT0 patients. DFS did not differ significantly between groups (5-year estimates: 78.7% vs. 78.9%; *p* = 0.860). OS also showed no significant difference (5-year estimates: 96.8% vs. 87.3%; *p* = 0.320). **Conclusions:** Residual DCIS was not associated with statistically significant differences in DFS or OS following NAC. However, these findings do not establish prognostic equivalence and should be interpreted cautiously given the limited number of ypTis patients and outcome events.

## Simple Summary

Pathological complete response after neoadjuvant chemotherapy is generally associated with favorable outcomes in breast cancer. However, some patients have residual ductal carcinoma in situ (DCIS) despite complete disappearance of invasive cancer. We compared patients with residual DCIS (ypTis) and no residual breast tumor (ypT0) among 175 node-negative patients achieving ypT0/is after neoadjuvant chemotherapy. Thirty-six patients had residual DCIS and 139 had no residual tumor. Although some clinical and MRI characteristics differed between groups, disease-free and overall survival did not differ significantly. These findings do not establish prognostic equivalence, and larger studies are needed.

## 1. Introduction

Among women, breast cancer is the most frequently diagnosed cancer and remains a leading cause of cancer-related mortality worldwide [1]. Neoadjuvant chemotherapy (NAC) is increasingly used in locally advanced and selected early-stage breast cancer to reduce tumor burden, assess treatment response, and facilitate surgical planning [2,3]. Pathological response varies according to patient characteristics and treatment strategies [4]. Pathological complete response (pCR) is an important prognostic marker associated with more favorable disease-free and overall survival, particularly in triple-negative and HER2-positive breast cancer, in which reported pCR rates range from approximately 35% to 71% [2,3,5,6,7,8]. In addition, a comprehensive meta-analysis found no significant difference in event-free survival between patients achieving pCR who did and did not receive additional adjuvant chemotherapy, supporting further evaluation of response-guided treatment de-escalation [5].

Ductal carcinoma in situ (DCIS) frequently coexists with invasive breast cancer, but its response to NAC and the prognostic significance of residual DCIS remain incompletely understood. In a retrospective cohort of 1148 patients, Labrosse et al. found that the presence of DCIS in pretreatment biopsy specimens was not significantly associated with pCR after NAC [9]. However, residual DCIS in the surgical specimen represents a distinct clinical question, particularly in patients without residual nodal disease. Therefore, the prognostic significance of residual DCIS compared with the absence of residual breast tumor in patients with ypN0 disease warrants further investigation.

NAC may also facilitate tumor downstaging and increase the feasibility of breast-conserving surgery, contributing to surgical de-escalation in appropriately selected patients [10]. However, the definition of pCR varies across studies, particularly regarding the presence of residual DCIS [11]. Accordingly, patients with no residual invasive disease but persistent DCIS may be classified as having pCR in some studies, whereas others classify them as ypTis. This distinction highlights the need to clarify the clinical significance of residual in situ disease.

The aim of this study was to evaluate residual DCIS after NAC and surgery, its associations with clinicopathological characteristics and radiological findings, and its relationship with clinical outcomes. We compared patients with ypTis and ypT0, focusing on molecular subtype, HER2-directed treatment, MRI findings, surgical approach, local recurrence, and survival outcomes.

## 2. Materials and Methods

### 2.1. Study Design and Population

A total of 831 consecutive patients with breast cancer diagnosed and treated at our institution between January 2019 and December 2023 were initially identified. Seventy-one patients had metastatic disease at initial presentation, and 213 were excluded because of primary DCIS, male breast cancer, bilateral breast cancer, or primary surgery without neoadjuvant chemotherapy. The remaining 547 patients received NAC followed by definitive surgery.

**Figure 1:**
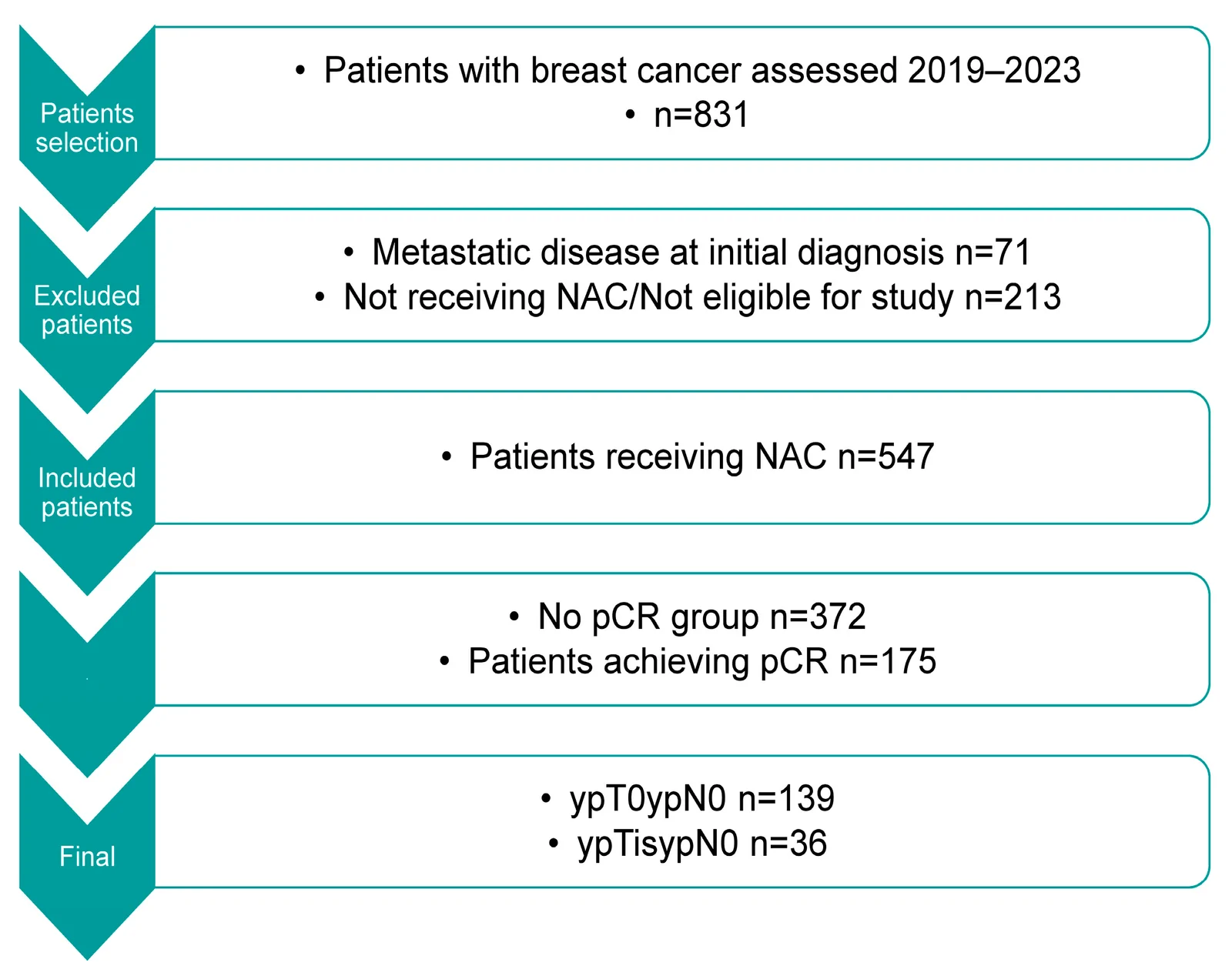
Flow diagram of patient selection.

Among these 547 patients, 175 achieved pCR, defined as ypT0/is ypN0, and constituted the final cohort: 139 patients with ypT0 ypN0 and 36 with ypTis ypN0. The remaining 372 patients had residual invasive breast disease and/or residual nodal disease and therefore did not meet the predefined eligibility criterion (Figure 1).

Patients were included consecutively according to the predefined criteria. The study period refers to diagnosis and treatment between 1 January 2019 and 31 December 2023. No patients were excluded solely because of missing MRI, pathological, or follow-up information.

Staging was performed using bilateral mammography, breast ultrasonography, breast magnetic resonance imaging (MRI), and 18F-FDG positron emission tomography/computed tomography (PET/CT). All patients received four cycles of anthracycline-based chemotherapy (adriamycin and cyclophosphamide), followed by either 12 weekly cycles of paclitaxel or four cycles of docetaxel. Among HER2-positive patients, some received dual HER2 blockade with pertuzumab, whereas others received trastuzumab alone because of financial constraints. All HER2-positive patients received adjuvant trastuzumab for a total duration of 52 weeks. Clinical response was assessed using physical examination, breast ultrasonography, and MRI. Surgical and axillary management was determined by a multidisciplinary tumor board.

Disease-free survival (DFS) was defined as the interval from definitive surgery to the first evidence of local recurrence, distant metastasis, or death from any cause, whichever occurred first. Overall survival (OS) was defined as the interval from diagnosis to death from any cause or last follow-up. The final follow-up was completed in December 2024. The median follow-up duration was 51 months (range, 10–69 months).

Pathological complete response (pCR) was defined as the absence of residual invasive carcinoma in the breast and regional lymph nodes, corresponding to ypT0/is ypN0 [12]. Within the pCR cohort, patients were classified according to the presence or absence of residual DCIS. ypT0 was defined as the absence of residual invasive or in situ carcinoma in the breast, with ypN0 status, whereas ypTis was defined as residual DCIS without residual invasive carcinoma in the breast, with ypN0 status. Thus, both ypT0 and ypTis were considered pCR categories while representing distinct pathological response categories.

### 2.2. Data Collection and Follow-Up

Clinical, pathological, treatment, MRI, and follow-up data were retrospectively extracted from the institutional clinical database and cross-checked against electronic medical records, pathology reports, radiology reports, operative records, and oncology follow-up records when required. The data were extracted and reviewed by the authors. In case of discrepancies, the original medical record or corresponding pathology/radiology report was considered the reference source. Recurrence was classified as local, regional, or distant based on available clinical, radiological, pathological, and operative documentation. No missing data were present for the variables included in the primary analyses; therefore, no imputation was performed.

### 2.3. Study Objective and Endpoints

The primary objective was to determine whether residual ypTis represented a distinct subgroup within the pCR population compared with ypT0. We hypothesized that residual ypTis may be associated with specific clinicopathological, radiological, and treatment-related characteristics despite complete eradication of the invasive tumor component.

The primary outcome was pathological response category (ypTis vs. ypT0). Secondary outcomes were DFS and OS. Clinicopathological, radiological, and treatment-related factors associated with residual ypTis were also evaluated.

### 2.4. Pathological Evaluation

For patients considered candidates for breast-conserving surgery, a localization clip was placed within the breast tumor before NAC to facilitate identification of the original tumor bed after treatment. Clipping was not routinely performed in patients planned for mastectomy. Following surgery, the tumor bed was identified using available clinical and radiological information, operative findings, and the clip when applicable. The identified region was sampled for histopathological evaluation of residual invasive carcinoma and DCIS. Histopathological evaluation was performed by the same breast pathologist throughout the study period.

Although extensive tumor-bed sampling was routine institutional practice, the number of tissue blocks or sections obtained from each tumor bed was not prospectively recorded. Therefore, the extent of sampling could not be quantified, and complete submission of the tumor bed could not be confirmed for every patient. The consistency of the sampling protocol throughout the study period could not be independently verified retrospectively.

Available pretreatment pathological records were retrospectively reviewed to identify concomitant DCIS at baseline. The nuclear grade of residual DCIS was also retrospectively assessed in patients with ypTis.

Although a numerical Miller–Payne grade was not routinely reported in the original pathology reports, complete absence of residual invasive carcinoma in the surgical specimen was considered consistent with Miller–Payne grade 5 for the invasive component [13].

Final staging and axillary lymph node response were assessed according to the American Joint Committee on Cancer (AJCC) Cancer Staging Manual, 8th Edition [14]. Axillary procedures included sentinel lymph node biopsy (SLNB) and axillary lymph node dissection (ALND), according to routine clinical practice. Only patients with pathologically confirmed ypN0 status and a breast response classified as ypT0 or ypTis were included in the study.

### 2.5. Immunohistochemical Assessment and Surrogate Molecular Subtyping

Estrogen receptor (ER) and progesterone receptor (PR) status were evaluated according to the American Society of Clinical Oncology/College of American Pathologists (ASCO/CAP) guidelines, using a 1% threshold for positivity [15,16]. HER2 expression was scored as 0, 1+, 2+, or 3+ according to membrane staining intensity and pattern. Cases with a score of 3+ were considered HER2-positive, whereas scores of 0 or 1+ were considered negative. For equivocal cases (2+), HER2 status was determined using Silver In Situ Hybridization (SISH); only cases with confirmed gene amplification were classified as HER2-positive [17].

The Ki-67 proliferation index was determined by evaluating at least 500 tumor cells in hotspot areas and was recorded as the percentage of positively stained nuclei [18]. A 20% cut-off was used according to the 2013 St. Gallen International Expert Consensus guidelines [19]. Based on ER, PR, HER2, and Ki-67 status, tumors were classified into five surrogate subtypes: Luminal A-like (ER+ and/or PR+, HER2−, Ki-67 < 20%), Luminal B-like/HER2-negative (ER+ and/or PR+, HER2−, Ki-67 ≥ 20%), Luminal B-like/HER2-positive (ER+ and/or PR+, HER2+), HR-negative/HER2-positive (ER−, PR−, HER2+), and triple-negative (ER−, PR−, HER2−).

### 2.6. Assessment of MRI Satellite Foci

Pretreatment breast MRI examinations were interpreted prospectively by two breast imaging radiologists as part of routine clinical care, with each examination assessed by one radiologist. MRI variables, including satellite foci, were retrospectively extracted from the original reports. Satellite foci were defined as separate suspicious enhancing foci distinct from the index lesion and explicitly reported as satellite foci, without predefined size or distance criteria. No blinded retrospective image review was performed, and inter-reader agreement could not be assessed. Satellite foci were considered imaging findings and were not assumed to represent pathologically confirmed DCIS. Enhancement kinetics were not prospectively recorded as a separate quantitative criterion.

### 2.7. Statistical Analyses

Continuous variables were expressed as mean ± standard deviation or median (range), as appropriate, and categorical variables as frequencies and percentages. Normality was assessed using the Kolmogorov–Smirnov test. Continuous variables were compared using the Mann–Whitney U test, and categorical variables using the Pearson chi-square or Fisher’s exact test, as appropriate.

DFS and OS were estimated using the Kaplan–Meier method and compared using the log-rank test. DFS was calculated from the date of definitive surgery, whereas OS was calculated from the date of initial breast cancer diagnosis. Kaplan–Meier estimates with 95% confidence intervals and numbers at risk were generated and verified using jamovi (version 2.6.44). Cox proportional hazards regression was used to estimate hazard ratios (HRs) with 95% confidence intervals for DFS and OS according to pathological response group (ypTis vs. ypT0).

Binary logistic regression was used to identify factors associated with residual ypTis. Univariable analyses were performed for prespecified clinical, pathological, treatment-related, and MRI-related variables, with odds ratios (ORs), 95% confidence intervals (CIs), and *p*-values reported.

Given the limited number of ypTis events (*n* = 36), the multivariable logistic regression model was restricted to two clinically relevant variables: HER2 status, and MRI-detected satellite foci. These variables were selected based on clinical relevance, biological plausibility, and statistically significant associations in the univariable analyses and were entered simultaneously into the final model. Adjusted ORs with 95% CIs and *p*-values were reported. Nagelkerke R^2^ was reported as a descriptive pseudo-R^2^ measure and was not interpreted as a measure of predictive accuracy. Given the exploratory nature of the analysis and limited number of ypTis events, the multivariable findings were interpreted as exploratory adjusted associations rather than as a validated prediction model.

An approximate detectable-effect analysis based on the Schoenfeld method for Cox regression was performed using the observed group allocation (36 ypTis and 139 ypT0 patients) and event counts. The calculations assumed a two-sided significance level of 0.05 and 80% power. Given the small number of local recurrence events (*n* = 4), a reliable detectable-effect estimate was not calculated. This was an approximate post hoc detectable-effect analysis rather than an a priori power calculation.

The primary survival analysis compared DFS and OS between patients with ypT0 ypN0 and ypTis ypN0. Comparisons of baseline clinicopathological characteristics, MRI findings, treatment characteristics, and subgroup analyses were considered exploratory. No formal multiplicity adjustment was applied because these analyses were exploratory rather than confirmatory.

All statistical analyses were performed using IBM SPSS Statistics for Windows, Version 26.0, and jamovi, version 2.6.44. All tests were two-sided, and a *p*-value < 0.05 was considered statistically significant.

## 3. Results

A total of 175 patients were included after NAC, including 139 (79.4%) with ypT0 and 36 (20.6%) with ypTis. The median age was 49 years (range, 28–75 years), with no significant difference between the ypTis and ypT0 groups (47 vs. 50 years, respectively; *p* = 0.450). When categorized as <50 versus ≥50 years, age distribution also did not differ significantly (*p* = 0.094). Median initial tumor diameter was 3.0 cm (range, 1.2–12.0 cm) in the ypT0 group and 3.35 cm (range, 1.2–12.0 cm) in the ypTis group (*p* = 0.165). Invasive ductal carcinoma, nuclear grade 3, and clinical N1 disease were predominant in both groups. Most patients underwent mastectomy. The distribution of clinical T stage differed significantly between groups (*p* = 0.014).

Among the 36 patients with ypTis, the median extent of residual DCIS was 0.3 cm (range, 0.1–1.3 cm). Residual DCIS was classified as NG2 in 11 patients (30.6%) and NG3 in 25 patients (69.4%).

Surgical margins were negative in all 139 patients with ypT0 and in 34 of 36 patients with ypTis (94.4%). Positive margins were observed in two patients with ypTis (5.6%); neither developed local recurrence.

Regarding axillary surgery, SLNB was performed in 21 patients with ypTis (58.3%) and 103 patients with ypT0 (74.1%), whereas ALND was performed in 15 patients with ypTis (41.7%) and 36 patients with ypT0 (25.9%). All patients included in the study had pathologically confirmed ypN0 status. No patients with isolated tumor cells in the axillary lymph nodes were identified.

HER2 positivity was more frequent in the ypTis group than in the ypT0 group (77.8% vs. 56.8%; *p* = 0.022). High proliferative activity (Ki-67 ≥ 20%) was observed in 91.7% of ypTis and 94.2% of ypT0 patients (*p* = 0.699). Adjuvant endocrine therapy was administered to 69.4% of ypTis and 56.1% of ypT0 patients (*p* = 0.147).

The distribution of neoadjuvant systemic therapy did not differ significantly between groups (*p* = 0.051). Among HER2-positive patients, trastuzumab alone was administered to 33 (41.8%) ypT0 and 14 (50.0%) ypTis patients, whereas trastuzumab plus pertuzumab was administered to 46 (58.2%) and 14 (50.0%), respectively (*p* = 0.451; Supplementary Table S1).

Pretreatment MRI demonstrated a significantly higher prevalence of satellite foci in the ypTis group than in the ypT0 group (61.1% vs. 38.1%; *p* = 0.013). Baseline characteristics are summarized in Table 1.

The overall distribution of immunohistochemical surrogate subtypes did not differ significantly between the ypTis and ypT0 groups (*p* = 0.146). In exploratory comparisons, luminal B-like/HER2-positive tumors were more frequent in the ypTis group than in the ypT0 group (55.6% vs. 36.7%; *p* = 0.056), whereas triple-negative tumors were more frequent in the ypT0 group (23.7% vs. 8.3%; *p* = 0.062). No significant differences were observed for luminal A-like (*p* = 0.582), luminal B-like/HER2-negative (*p* = 0.698), or HR-negative/HER2-positive (*p* = 0.783) subtypes (Table 2). These comparisons were considered exploratory.

**Figure 2:**
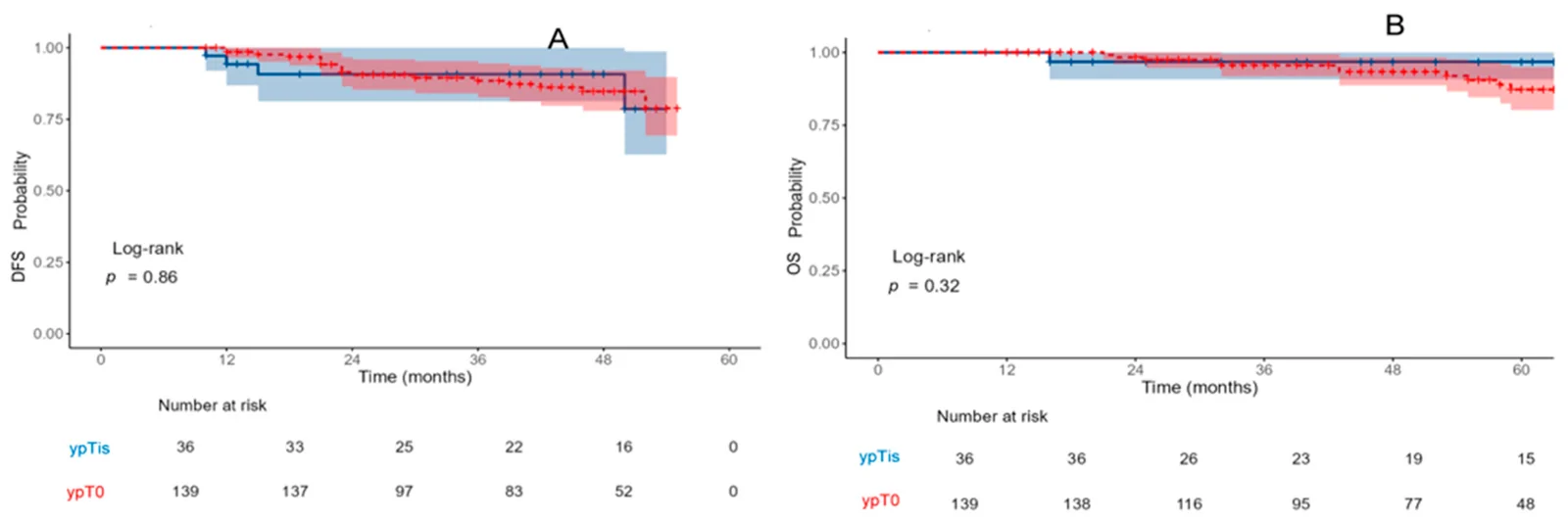
(**A**) Disease-free survival (DFS) probability in the ypTis and ypT0 groups; (**B**) overall survival (OS) probability in the ypTis and ypT0 groups. Blue indicates ypTis, and red indicates ypT0.

The median follow-up duration was 51 months (range, 10–69 months). During follow-up, 23 DFS events and 12 deaths were observed. Median DFS and OS were not reached in either group. Kaplan–Meier analysis showed no significant difference in DFS between the ypTis and ypT0 groups (log-rank *p* = 0.860). The 1-, 3-, and 5-year DFS probabilities were 94.3%, 90.8%, and 78.7% in the ypTis group and 98.5%, 88.5%, and 78.9% in the ypT0 group, respectively (Figure 2A). The corresponding HR was 0.91 (95% CI, 0.34–2.47).

Similarly, no significant difference in OS was observed between the groups (log-rank *p* = 0.320). The 1-, 3-, and 5-year OS probabilities were 96.8%, 96.8%, and 96.8% in the ypTis group and 100.0%, 95.5%, and 87.3% in the ypT0 group, respectively (Figure 2B). The corresponding HR was 2.69 (95% CI, 0.35–20.87).

In the Luminal A-like subgroup, the overall DFS analysis yielded a statistically significant log-rank result (*p* = 0.027; Supplementary Figure S1A). However, this subgroup included only four patients, with no patients classified as ypTis; therefore, a comparison of DFS between the ypT0 and ypTis groups was not possible. In the other evaluable immunohistochemical surrogate subtypes, no statistically significant differences in DFS or OS were observed between the ypT0 and ypTis groups (Table 3). The corresponding subgroup-specific Kaplan–Meier curves for DFS and OS are presented in Supplementary Figures S1 and S2, respectively. These findings should be interpreted in light of the small subgroup sizes and limited number of outcome events.

Based on the observed 23 DFS events and 12 OS deaths, the estimated detectable hazard ratios were approximately 4.24 (or 0.24 in the opposite direction) for DFS and 7.39 (or 0.14 in the opposite direction) for OS, assuming a two-sided significance level of 0.05 and 80% power. The results are presented in Supplementary Table S3, Part A.

Among patients undergoing breast-conserving surgery, local recurrence occurred in 2 of 13 (15.4%) ypTis patients and 1 of 63 (1.6%) ypT0 patients (*p* = 0.074). Among patients undergoing mastectomy, local recurrence occurred in 1 of 23 (4.3%) ypTis patients and none of 76 ypT0 patients (*p* = 0.232). These analyses were considered descriptive because of the small number of events.

Overall, local recurrence occurred in 3/36 (8.3%) patients with ypTis and 1/139 (0.7%) patients with ypT0 (*p* = 0.028, Fisher’s exact test). Among the three patients with ypTis who developed local recurrence, two had undergone breast-conserving surgery (BCS) and one had undergone mastectomy. The patient with ypT0 who developed local recurrence had undergone BCS. All three patients who underwent BCS received whole-breast and regional nodal radiotherapy (50 Gy) with a 10-Gy tumor-bed boost. The patient with ypTis who underwent mastectomy had negative surgical margins. The patient with ypT0 developed recurrence at the surgical incision line 12 months after surgery and subsequently underwent excision followed by chest-wall radiotherapy with an electron boost.

Binary logistic regression was performed to identify factors associated with residual ypTis. In univariable analysis, HER2-positive status (OR, 2.658; 95% CI, 1.131–6.247; *p* = 0.025), and MRI-detected satellite foci (OR, 2.550; 95% CI, 1.202–5.411; *p* = 0.015) were significantly associated with residual ypTis. The complete results of the univariable logistic regression analyses are provided in Supplementary Table S2. In multivariable logistic regression analysis, HER2 positivity (adjusted OR, 2.50; 95% CI, 1.05–5.93; *p* = 0.038) and MRI-detected satellite foci (adjusted OR, 2.41; 95% CI, 1.12–5.16; *p* = 0.024) were independently associated with residual ypTis (Table 4). In a sensitivity analysis excluding the two patients with ypTis and positive surgical margins, HER2 positivity (OR 2.74, 95% CI 1.10–6.82, *p* = 0.030) and MRI-detected satellite foci (OR 2.82, 95% CI 1.27–6.23, *p* = 0.011) remained independently associated with ypTis (Supplementary Table S3, Part B).

## 4. Discussion

The prognostic significance of residual ductal carcinoma in situ (DCIS), classified as ypTis, following neoadjuvant chemotherapy (NAC) remains an area of clinical interest. In the present study, we evaluated clinical characteristics, treatment patterns, recurrence patterns, and survival outcomes in patients with ypTis compared with those achieving ypT0 after NAC. The proportion of patients with ypTis was 20.6% (36/175) within our selected ypT0/is ypN0 population. Reported frequencies of residual ypTis vary across studies, reflecting differences in patient selection, treatment regimens, pathological definitions, sampling methods, and study denominators [20,21,22,23,24,25].

The prognostic significance of ypTis remains controversial. Mazouni et al. [20] suggested that residual DCIS did not adversely affect survival among patients with complete eradication of invasive disease, whereas the pooled analysis by von Minckwitz et al. [21] reported worse disease-free survival in patients with residual DCIS. More recently, the I-SPY2 trial [22] found no statistically significant difference in outcomes according to the presence of residual DCIS among patients achieving pCR. Similarly, no statistically significant differences in DFS or OS were observed between ypTis and ypT0 in our cohort. These findings support the possibility that residual in situ disease may have a different prognostic significance from residual invasive disease [20,22]. However, the small ypTis subgroup and limited number of outcome events preclude conclusions regarding prognostic equivalence.

Neoadjuvant systemic therapy differed numerically between pathological response groups. Chemotherapy alone was more frequent in the ypT0 group, whereas chemotherapy combined with trastuzumab, with or without pertuzumab, was more frequent in the ypTis group; however, the overall difference did not reach statistical significance (*p* = 0.051). The multicenter NEOPERSUR study reported substantial pathological complete response rates with neoadjuvant trastuzumab and pertuzumab combined with chemotherapy in patients with HER2-positive early-stage breast cancer [26]. Although that study did not specifically investigate residual DCIS, it provides relevant context regarding pathological response to HER2-targeted treatment. In our cohort, the observed treatment distribution may partly reflect the higher prevalence of HER2-positive disease in the ypTis group rather than a differential effect of specific regimens. Accordingly, no causal inference regarding the effect of HER2-targeted treatment on residual ypTis can be made.

The biological response of DCIS to neoadjuvant systemic therapy may differ from that of the invasive component. In a systematic review, Wazir et al. reported complete eradication of DCIS in 40.5% of patients with documented pretreatment DCIS, with similar eradication rates in triple-negative and HER2-positive disease (45% and 46%, respectively) [27]. In our cohort, HER2 positivity was independently associated with residual ypTis. However, because baseline DCIS status was not systematically documented, we could not determine whether this association reflected the persistence of pre-existing DCIS or differences in the response of invasive and in situ components.

The biological basis of residual DCIS after neoadjuvant treatment remains uncertain. Mazouni et al. [21] suggested that residual DCIS may reflect differential treatment sensitivity between invasive and in situ components. In our cohort, the association between HER2 positivity and residual ypTis may reflect greater eradication of the invasive component with HER2-directed treatment while small residual in situ components remain detectable. Labrosse et al. reported that the presence of an in situ component on pretreatment biopsy was not significantly associated with pathological response to NAC [9]. However, because baseline DCIS status was not systematically documented in our cohort, we could not reliably assess this association or determine whether residual DCIS represented persistence of a pre-existing component.

In the nationwide study by Ploumen et al. [23], 881 patients had residual DCIS without invasive disease (ypTis) and 5847 achieved ypT0 among 20,495 patients treated with neoadjuvant systemic therapy. Among patients with either ypTis or ypT0 breast response, residual DCIS accounted for approximately 13.1%. In our cohort, which was restricted to patients with ypT0/is and ypN0 status, ypTis accounted for 20.6%. These proportions should be interpreted cautiously because the study populations differed in their eligibility criteria, particularly with respect to nodal status, as well as in patient selection and potentially treatment and pathological assessment. Therefore, the observed difference in ypTis frequency should not be interpreted as a direct between-study comparison.

Shin et al. [25] reported significantly worse distant metastasis-free survival among patients with ypTis and HR-negative breast cancer, whereas no significant difference was observed among HR-positive patients. In our cohort, no statistically significant differences in DFS or OS were observed between ypTis and ypT0 across the examined molecular subgroups. However, the small number of HR-negative patients with ypTis limits the statistical power of these subgroup analyses; therefore, our findings should be interpreted with caution and should not be taken as evidence against a potential prognostic role of ypTis in HR-negative disease. Conversely, Wang et al. [28], in a retrospective study of patients undergoing breast-conserving therapy after NAC, reported better long-term survival and lower recurrence rates among patients achieving ypT0 than among those with ypTis or residual invasive disease. Outcomes among patients with ypTis were comparable to those among patients who did not achieve pCR, suggesting that the prognostic implications of pathological response may vary according to residual disease subtype.

HER2 positivity was observed in 77.8% of patients with ypTis in our cohort. In the I-SPY2 analysis, 48 of 70 patients with pCR and residual DCIS were HER2-positive (68.6%) [22]. These figures describe the proportion of patients with residual DCIS who were HER2-positive, rather than the proportion of HER2-positive patients who developed residual DCIS. Differences in patient selection, treatment regimens, and HER2-directed therapy may account for the difference between these proportions. Groen et al. reported that DCIS was eradicated after neoadjuvant systemic therapy in 46% of patients with HER2-positive invasive breast cancer and associated DCIS [29], suggesting that the response of the in situ component may vary even in HER2-positive disease. In our exploratory multivariable logistic regression analysis, HER2 positivity and MRI-detected satellite foci were independently associated with residual ypTis. These associations require validation in larger cohorts.

The ypTis group had a higher proportion of cT4 tumors than the ypT0 group (13.9% vs. 2.2%), alongside a significant difference in the overall clinical T-stage distribution (*p* = 0.014). ER and PR positivity also tended to be more frequent in the ypTis group, although these differences did not reach statistical significance (*p* = 0.058 and *p* = 0.067, respectively). These trends should be interpreted cautiously because hormone receptor status, HER2 status, and molecular subtype are biologically interrelated. The observed enrichment of HER2-positive and Luminal B HER2-positive tumors in the ypTis group may therefore reflect overlapping tumor characteristics rather than independent associations.

The residual in situ lesions in our cohort were generally small, with a median diameter of 0.3 cm (range, 0.1–1.3 cm). Although extensive tumor-bed sampling was routine institutional practice, patient-level sampling data were unavailable, preventing quantification of its contribution to the observed ypTis rate. Differences in pathological sampling may partly explain variations in ypTis rates across studies. Importantly, residual DCIS identified in surgical specimens does not establish that MRI-detected satellite foci represented pathologically confirmed DCIS.

Regarding distant failure, Acevedo et al. [30] reported that some patients who subsequently developed metastatic disease had previously achieved pCR, highlighting that pCR does not eliminate the risk of distant recurrence. In our cohort, six patients in the ypT0 group (4.3%) developed brain metastasis during follow-up, with most cases occurring in patients with triple-negative breast cancer. Direct numerical comparison is not appropriate because of differences in denominators and clinical questions. Overall, local recurrence occurred in 4/175 patients (2.3%) in our cohort, although differences in cohort selection, definitions, treatment characteristics, and follow-up limit direct comparison with previous studies [25,30].

The crude local recurrence rate was higher in the ypTis group than in the ypT0 group (3/36 [8.3%] vs. 1/139 [0.7%]; *p* = 0.028). However, only four local recurrence events occurred overall. Two of the three ypTis recurrences occurred after breast-conserving surgery, whereas the only recurrence after mastectomy occurred in a patient who had declined adjuvant radiotherapy. After stratification by surgical approach, the difference remained numerically apparent among patients undergoing breast-conserving surgery (15.4% vs. 1.6%; *p* = 0.074), but was not statistically significant; no significant difference was observed among patients undergoing mastectomy (4.3% vs. 0%; *p* = 0.232). Given the small number of events, the higher crude recurrence rate cannot be attributed to ypTis alone, and the potential influence of surgical and radiotherapy-related factors must be considered.

Our findings regarding tumor characteristics and clinical outcomes are consistent with the study by Tinterri et al. [31]. They reported multifocal disease in 62.5% of the ypTis group compared with 27.8% of the ypT0 group (*p* = 0.006), paralleling the higher prevalence of satellite foci on baseline MRI in our ypTis group (61.1% vs. 38.1%, *p* = 0.013). Although Tinterri et al. assessed multifocality pathologically, whereas satellite foci in our study were identified on pre-treatment MRI, both findings may reflect greater tumor focality in patients with residual in situ disease. Similar to their findings, triple-negative breast cancer was more prevalent among patients achieving ypT0 in our cohort. Importantly, neither study demonstrated a statistically significant difference in DFS or OS between ypTis and ypT0 [30].

Several limitations should be acknowledged. First, the retrospective, single-center design and restriction to patients with ypN0 disease may have introduced selection bias and limit the generalizability of our findings to patients with residual nodal disease. Second, the ypTis subgroup comprised only 36 patients, with few outcome events and small molecular subgroups. Only three patients with triple-negative breast cancer had ypTis, and no patients with Luminal A-like disease had ypTis. Consequently, subgroup comparisons may have been underpowered, and the limited number of events precluded robust adjusted modeling of survival and local recurrence. Third, the median follow-up of 51 months may be insufficient to assess long-term outcomes, particularly in hormone receptor-positive disease. Fourth, baseline DCIS status was not systematically documented, preventing reliable distinction between persistent pre-existing DCIS and residual DCIS identified after treatment. Fifth, MRI-detected satellite foci were extracted retrospectively from routine radiology reports rather than assessed through standardized, independent image rereading. Reader and reporting variability therefore cannot be excluded, and these foci cannot be assumed to represent pathologically confirmed DCIS. Similarly, although extensive tumor-bed sampling was routine institutional practice, patient-level sampling data were unavailable, and complete submission of the tumor bed could not be confirmed for every patient.

The limited number of outcome events constrained the statistical power of the study. The approximate detectable-effect analysis suggested that the study was primarily capable of detecting large effects for DFS and OS and may have lacked sufficient power to identify more modest but clinically meaningful differences. Furthermore, only four local recurrence events were observed, precluding a reliable assessment of this outcome. Therefore, the findings, particularly those related to OS and local recurrence, should be interpreted cautiously. Finally, differences in neoadjuvant and adjuvant treatments, including HER2-directed therapy, endocrine therapy, surgery, and radiotherapy, may have influenced recurrence outcomes. Such differences, together with variations in treatment era and regimen, should be considered when comparing pathological response across cohorts.

## 5. Conclusions

Residual ypTis after neoadjuvant chemotherapy was associated with specific tumor characteristics, particularly HER2 positivity and MRI-detected satellite foci. No statistically significant differences in DFS or OS were observed between patients with ypTis and ypT0 during the available follow-up period. However, these findings should be interpreted cautiously given the limited number of ypTis patients and outcome events and should not be interpreted as evidence of prognostic equivalence or used as a basis for changing clinical management.

## Figures and Tables

**Table 1:** Baseline characteristics of the study population.

	ypT0 (*n*, %)		ypTis (*n*, %)		*p* Value
	*n* = 139	79.4%	*n* = 36	20.6%	
Age					
<50 years	71	51.1	24	66.7	
≥50 years	68	48.9	12	33.3	0.094
Tumor diameter (cm)					
median	3.0		3.35		
(range)	(1.2–12.0)		(1.2–12.0)		0.165 ^m^
Histology					
IDC	123	88.5	32	88.9	
Others	16	11.5	4	11.1	0.946
Nuclear grade					
2	19	13.7	5	13.9	
3	120	86.3	31	86.1	0.973
cT stage					
1	14	10.1	6	16.7	
2	105	75.5	20	55.6	
3	17	12.2	5	13.9	
4	3	2.2	5	13.9	0.014 ^f^
cN stage					
0	19	13.7	5	13.9	
1	74	53.2	18	50.0	
2	38	27.3	11	30.6	
3	8	5.8	2	5.6	0.983
cStage					
II	81	58.3	21	58.3	
III	58	41.7	15	41.7	0.995
Operation type					
BCS	63	45.3	13	36.1	
Mastectomy	76	54.7	23	63.9	0.320
Estrogen receptor					
positive	72	51.8	25	69.4	
negative	67	48.2	11	30.6	0.058
Progesterone receptor					
positive	65	46.8	23	63.9	
negative	74	53.2	13	36.1	0.067
HER2 status					
positive	79	56.8	28	77.8	
negative	60	43.2	8	22.2	0.022
Neoadjuvant systemic therapy					
chemotherapy alone	60	43.2	8	22.2	
chemotherapy + T	33	23.7	14	38.9	
chemotherapy + T+P	46	33.1	14	38.9	0.051
Ki-67					
<20	8	5.8	3	8.3	
≥20	131	94.2	33	91.7	0.699 ^f^
Adjuvant endocrine therapy					
present	78	56.1	25	69.4	0.147
absent	61	43.9	11	30.6	
1st MRI satellite foci					
present	53	38.1	22	61.1	
absent	86	61.9	14	38.9	0.013

IDC, invasive ductal carcinoma; cT, clinical tumor size stage; cN, clinical lymph node status; cStage, Clinical tumor stage; BCS, Breast Conserving Surgery; HER2, Human Epidermal growth factor Receptor 2; MRI, magnetic resonance imaging, T: trastuzumab, P: pertuzumab; ^m^: Mann–Whitney U test; ^f^: Fisher’s exact test). Footnote: HR-positive was defined as ER-positive and/or PR-positive. ER and PR status overlapped; 82 patients (46.9%) were positive for both ER and PR.

**Table 2:** Immunohistochemical surrogate subtype distribution by ypT0 and ypTis status.

	ypT0		ypTis		*p*
	*n*	(%)	*n*	(%)	
Luminal A-like	4	(2.9)	0	(0)	0.582 ^f^
Luminal B-like/ HER2-	23	(16.5)	5	(13.9)	0.698
Luminal B-like/HER2+	51	(36.7)	20	(55.6)	0.056 ^f^
HR-negative/HER2 positive	28	(20.1)	8	(22.2)	0.783
Triple-negative	33	(23.7)	3	(8.3)	0.062 ^f^
Overall subtype	139	(79)	36	(21)	0.146 ^f^

*p*-values were calculated using the chi-square test or Fisher’s exact test, as appropriate. Superscript “^f^” indicates comparisons performed using Fisher’s exact test because of small expected cell counts.

**Table 3:** Comparison of disease-free survival (DFS) and overall survival (OS) between ypT0 and ypTis groups by immunohistochemical surrogate subtype.

	DFS (ypT0 vs. ypTis) *p*	OS (ypT0 vs. ypTis) *p*
Luminal A-like	NE	NE
Luminal B-like HER2-	0.504	0.539
Luminal B-like HER2+	0.812	0.906
HR-negative/HER2 positive	0.360	0.617
Triple-negative	0.483	0.447
All cohort	0.861	0.328

*p*-values were calculated using the log-rank (Mantel–Cox) test. The Luminal A-like subgroup was not estimable because no patients with ypTis were present in this subgroup. Subgroups with very few events did not permit reliable hazard-ratio estimation. NE, not estimable.

**Table 4:** Univariable and multivariable logistic regression analysis of factors associated with residual ypTis.

Predictor	Univariable OR	95%CI	*p* Value	Multivariable OR	95%CI	*p* Value
HER2 positive vs. negative	2.658	1.131–6.247	0.025	2.496	1.050–5.929	0.038
MRI satellite foci, present vs. absent	2.55	1.202–5.411	0.015	2.408	1.123–5.155	0.024

The multivariable model included HER2 status and MRI-detected satellite foci, entered simultaneously. Reference categories were HER2-negative status and absence of MRI-detected satellite foci. The model included all 175 patients and had a Nagelkerke R^2^ of 0.094. Given the limited number of ypTis events (*n* = 36), the multivariable analysis was considered exploratory, and the estimates represent adjusted associations rather than a validated prediction model.

## Data Availability

The datasets analyzed during the current study are not publicly available as the personal information of patients must be protected but are available from the corresponding author upon reasonable request.

## Supplementary Materials

The following supporting information can be downloaded at https://www.mdpi.com/article/10.3390/cancers18213491/s1, Figure S1. DFS for all subgroups; Figure S2. OS for subtypes; Table S1. Distribution of HER2-targeted therapy among HER2-positive patients according to pathological response; Table S2. Univariable logistic regression analysis of clinicopathological and imaging factors associated with residual ypTis; Table S3. part A. Approximate detectable hazard ratios for survival outcomes, part B. Sensitivity analysis of factors associated with ypTis after excluding the two patients with positive surgical margins.

## Abbreviations

The following abbreviations are used in this manuscript:

ASCO/CAP	American Society of Clinical Oncology/College of American Pathologists
BCS	Breast Conserving Surgery
cN	Clinical nodal stage
cT	Clinical tumor stage
DCIS	Ductal Carcinoma In Situ
DFS	Disease-free Survival
ER	Estrogen Receptor
HER2	Human Epidermal growth factor Receptor 2
HR	Hormone receptor
IDC	Invasive Ductal Carcinoma
Ki-67	Ki-67 proliferation index
MRI	Magnetic Resonance Imaging
NAC	Neoadjuvant Chemotherapy
OS	Overall Survival
pCR	Pathological Complete Response
P	Pertuzumab
PR	Progesterone Receptor
SISH	Silver-enhanced In Situ Hybridization
T	Trastuzumab
ypN0	No residual regional nodal disease
ypT0	No residual invasive or in situ carcinoma
ypTis	Residual ductal carcinoma in situ without residual invasive carcinoma
