# Supplementary material for: Residual DCIS Versus No Residual Breast Tumor After Neoadjuvant Chemotherapy in ypN0 Breast Cancer: A Retrospective Clinical, MRI, and Survival Comparison
Source: Cancers (Basel). 2026 Oct 29;18(21):3491. doi: 10.3390/cancers18213491 (PMC13761685; doi:10.3390/cancers18213491)
Supplement: Supplementary file 1 [file cancers-18-03491-s001.zip › Supp.Table S1.The distribution of NAC.pdf]

Supplementary Table S1. Distribution of HER2-targeted therapy among HER2-positive patients according to pathological response.

**HER2-targeted therapy**	**ypT0 (n=79)**	**ypTis (n=28)**	**p**
Trastuzumab	33 (41.8%)	14 (50.0%)	
Trastuzumab + pertuzumab	46 (58.2%)	14 (50.0%)	0.451
