# Supplementary material for: Residual DCIS Versus No Residual Breast Tumor After Neoadjuvant Chemotherapy in ypN0 Breast Cancer: A Retrospective Clinical, MRI, and Survival Comparison
Source: Cancers (Basel). 2026 Oct 29;18(21):3491. doi: 10.3390/cancers18213491 (PMC13761685; doi:10.3390/cancers18213491)
Supplement: Supplementary file 1 [file cancers-18-03491-s001.zip › Supp.Table S2.Univariate log regression analysis.pdf]

Supplementary Table S2. Univariable logistic regression analysis of clinicopathological and imaging factors associated with residual ypTis

Variable	OR	95% CI	p value
Age	0.984	0.951–1.020	0.355
Tumor diameter	1.146	0.998–1.316	0.054
**cT stage**			
2 vs 1	0.444	0.153–1.290	0.137
3 vs 1	0.686	0.172–2.730	0.593
4 vs 1	3.889	0.695–21.750	0.122
**cN stage**			
1 vs 0	0.924	0.304–2.809	0.890
2 vs 0	1.100	0.334–3.623	0.875
3 vs 0	0.950	0.152–5.959	0.956
ER positive vs negative	2.115	0.966–4.629	0.061
PR positive vs negative	2.014	0.945–4.295	0.070
HER2 positive vs negative	2.658	1.131–6.247	**0.025**
Ki-67 ≥20% vs <20%	1.041	0.325–3.328	0.946
Postmenopausal vs premenopausal	0.479	0.222–1.033	0.060
MRM vs MKC	1.467	0.687–3.129	0.322
MRI satellite foci present vs absent	2.550	1.202–5.411	**0.015**
Adjuvant endocrine therapy present vs absent	1.777	0.811–3.894	0.151
Radiotherapy, yes vs no	1.037	0.112–9.570	0.974
