# Supplementary material for: Residual DCIS Versus No Residual Breast Tumor After Neoadjuvant Chemotherapy in ypN0 Breast Cancer: A Retrospective Clinical, MRI, and Survival Comparison
Source: Cancers (Basel). 2026 Oct 29;18(21):3491. doi: 10.3390/cancers18213491 (PMC13761685; doi:10.3390/cancers18213491)
Supplement: Supplementary file 1 [file cancers-18-03491-s001.zip › Supp.Table S3.Detectable HRs and Sensitivity analysis.pdf]

Supplementary Table S3, part A. Approximate detectable hazard ratios for survival outcomes

Outcome	Total patients	Number of events	Detectable HR (80% power)
**Disease-free survival (DFS)**	175	23	0.236–4.243
**Overall survival (OS)**	175	12	0.135–7.395

(Estimates were calculated using the Schoenfeld approximation, assuming a two-sided α of 0.05, 80% power, and the observed group allocation. These estimates are approximate and do not fully account for the distribution of events between groups. They represent detectable-effect estimates based on the observed event counts, not an a priori power calculation.)

Supplementary Table S3, part B. Sensitivity analysis of factors associated with ypTis after excluding the two patients with positive surgical margins.

Predictor	Multivariable OR (main)	95%CI	p value	After 2 patients out (OR)	95%CI	p value
HER2 positive vs negative	2.496	1.050-5.929	**0.038**	2.744	1.103-6.823	**0.030**
MRI satellite foci, present vs absent	2.408	1.123-5.155	**0.024**	2.816	1.273-6.227	**0.011**

(Note: OR, odds ratio; CI, confidence interval. The sensitivity analysis excluded two patients with ypTis and positive surgical margins. The main and sensitivity analyses used the same multivariable logistic regression model).
