# Supplementary material for: Residual DCIS Versus No Residual Breast Tumor After Neoadjuvant Chemotherapy in ypN0 Breast Cancer: A Retrospective Clinical, MRI, and Survival Comparison
Source: Cancers (Basel). 2026 Oct 29;18(21):3491. doi: 10.3390/cancers18213491 (PMC13761685; doi:10.3390/cancers18213491)
Supplement: Supplementary file 1 [file cancers-18-03491-s001.zip › Supplementary Figure-S1A1B1C1D1E. DFS for all subgroups.pdf]

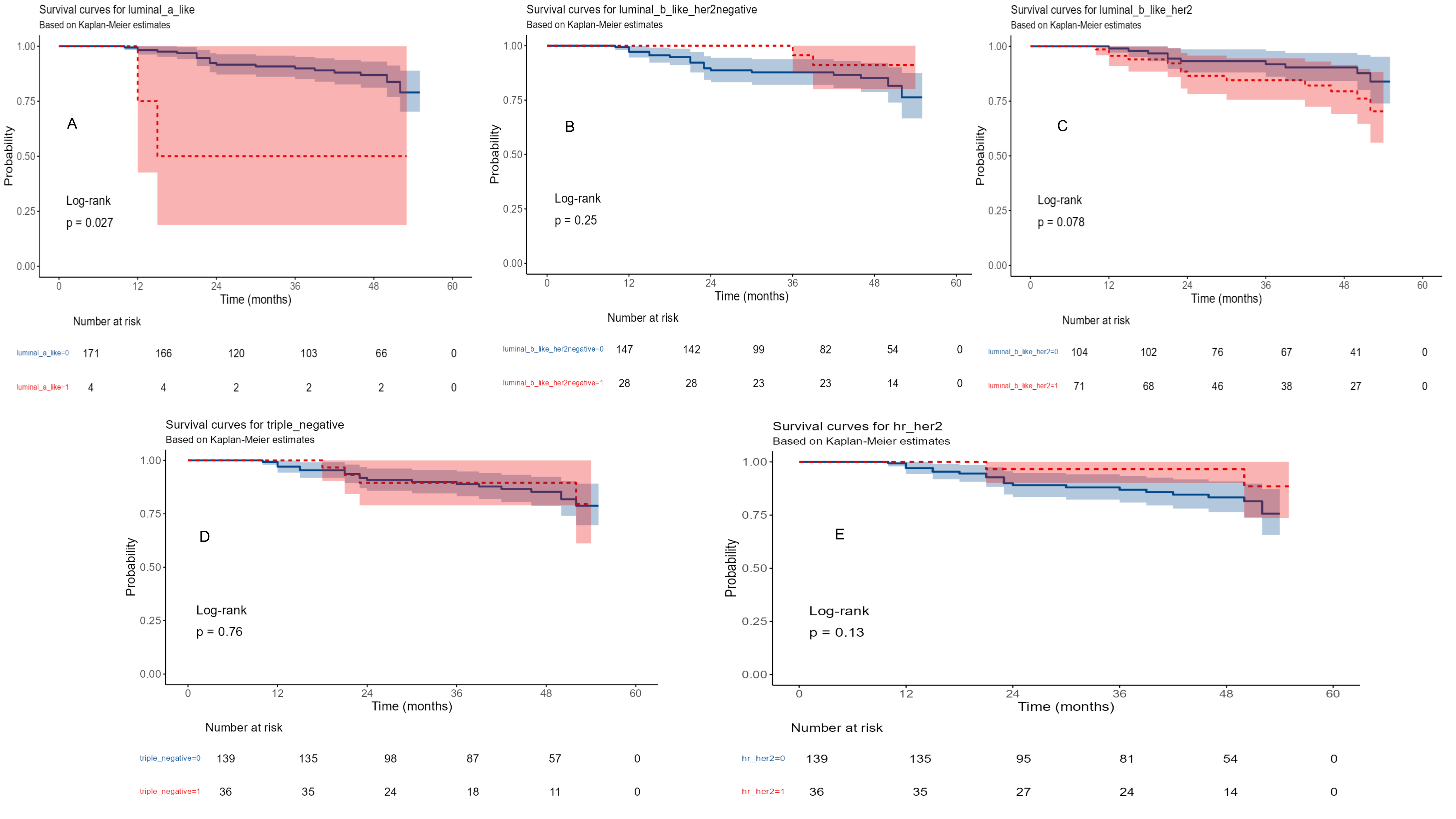
Survival curves for luminal_a_like
Based on Kaplan-Meier estimates
A
Log-rank
p = 0.027
Probability
1.00
0.75
0.50
0.25
0.00
Time (months)
0
12
24
36
48
60
Number at risk
luminal_a_like=0 171 166 120 103 66 0
luminal_a_like=1 4 4 2 2 2 0
Survival curves for luminal_b_like_her2negative
Based on Kaplan-Meier estimates
B
Log-rank
p = 0.25
Number at risk
luminal_b_like_her2negative=0 147 142 99 82 54 0
luminal_b_like_her2negative=1 28 28 23 23 14 0
Survival curves for luminal_b_like_her2
Based on Kaplan-Meier estimates
C
Log-rank
p = 0.078
Number at risk
luminal_b_like_her2=0 104 102 76 67 41 0
luminal_b_like_her2=1 71 68 46 38 27 0
Survival curves for triple_negative
Based on Kaplan-Meier estimates
D
Log-rank
p = 0.76
Number at risk
triple_negative=0 139 135 98 87 57 0
triple_negative=1 36 35 24 18 11 0
Survival curves for hr_her2
Based on Kaplan-Meier estimates
E
Log-rank
p = 0.13
Number at risk
hr_her2=0 139 135 95 81 54 0
hr_her2=1 36 35 27 24 14 0
