# Supplementary material for: Residual DCIS Versus No Residual Breast Tumor After Neoadjuvant Chemotherapy in ypN0 Breast Cancer: A Retrospective Clinical, MRI, and Survival Comparison
Source: Cancers (Basel). 2026 Oct 29;18(21):3491. doi: 10.3390/cancers18213491 (PMC13761685; doi:10.3390/cancers18213491)
Supplement: Supplementary file 1 [file cancers-18-03491-s001.zip › Supplementary Figure-S2A2B2C2D2E. OS for subtypes.pdf]

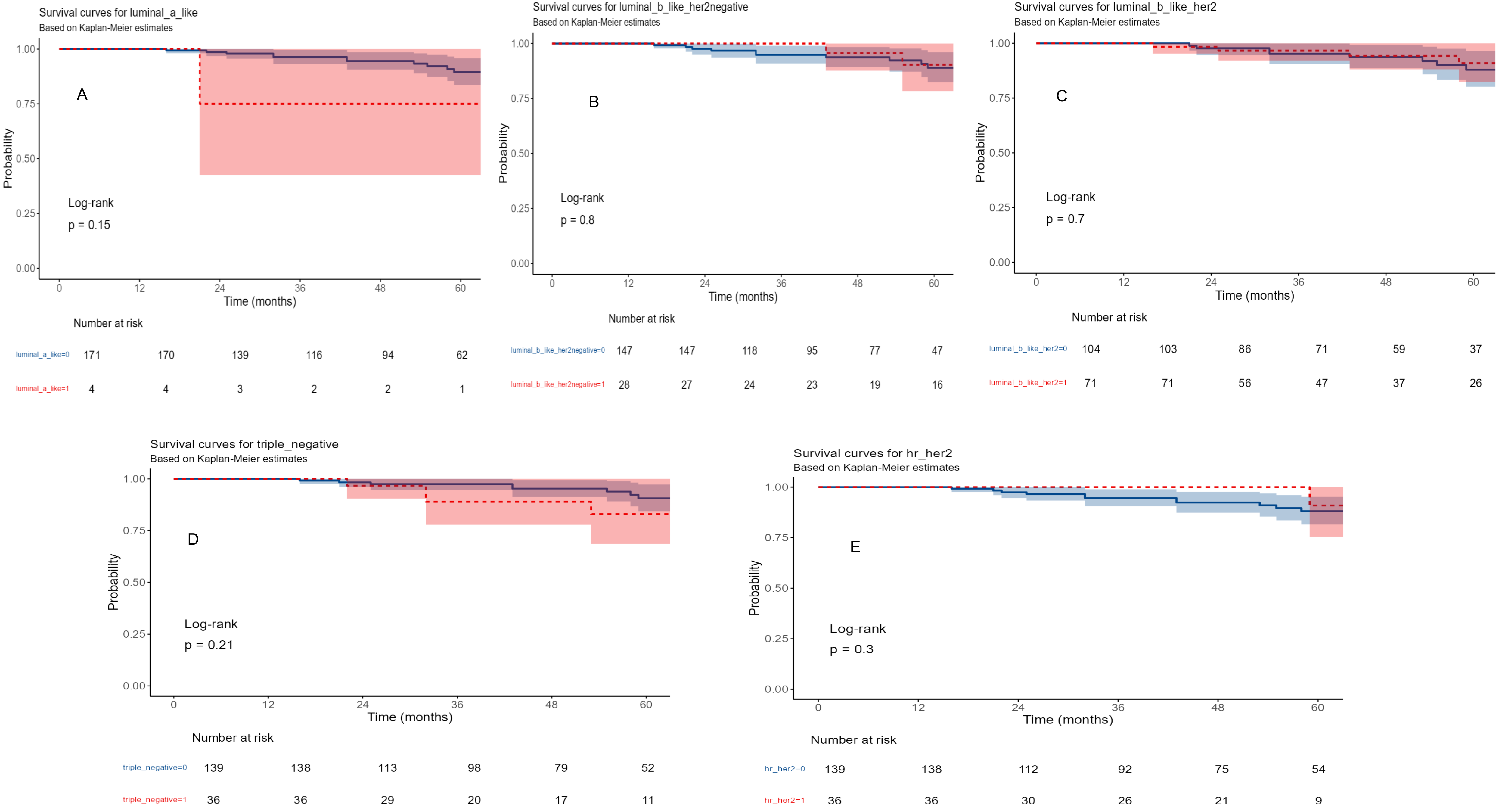

Survival curves for luminal_a_like
Based on Kaplan-Meier estimates
A
Log-rank
p = 0.15
Probability
1.00
0.75
0.50
0.25
0.00
0
12
24
36
48
60
Time (months)
Number at risk
luminal_a_like=0 171 170 139 116 94 62
luminal_a_like=1 4 4 3 2 2 1
Survival curves for luminal_b_like_her2negative
Based on Kaplan-Meier estimates
B
Log-rank
p = 0.8
Probability
1.00
0.75
0.50
0.25
0.00
0
12
24
36
48
60
Time (months)
Number at risk
luminal_b_like_her2negative=0 147 147 118 95 77 47
luminal_b_like_her2negative=1 28 27 24 23 19 16
Survival curves for luminal_b_like_her2
Based on Kaplan-Meier estimates
C
Log-rank
p = 0.7
Probability
1.00
0.75
0.50
0.25
0.00
0
12
24
36
48
60
Time (months)
Number at risk
luminal_b_like_her2=0 104 103 86 71 59 37
luminal_b_like_her2=1 71 71 56 47 37 26
Survival curves for triple_negative
Based on Kaplan-Meier estimates
D
Log-rank
p = 0.21
Probability
1.00
0.75
0.50
0.25
0.00
0
12
24
36
48
60
Time (months)
Number at risk
triple_negative=0 139 138 113 98 79 52
triple_negative=1 36 36 29 20 17 11
Survival curves for hr_her2
Based on Kaplan-Meier estimates
E
Log-rank
p = 0.3
Probability
1.00
0.75
0.50
0.25
0.00
0
12
24
36
48
60
Time (months)
Number at risk
hr_her2=0 139 138 112 92 75 54
hr_her2=1 36 36 30 26 21 9
